# Influence of Solvents and Halogenation on ESIPT of Benzimidazole Derivatives for Designing Turn-on Fluorescence Probes

**DOI:** 10.1021/acsomega.4c00488

**Published:** 2024-05-10

**Authors:** Murillo H. Queiroz, Tiago V. Alves, Roberto Rivelino, Sylvio Canuto

**Affiliations:** † Departamento de Físico-Química, Instituto de Química, Universidade Federal da Bahia, Rua Barão de Jeremoabo, 147, Salvador, Bahia 40170-115, Brazil; ‡ Instituto de Física, Universidade Federal da Bahia, Salvador, Bahia 40210-340, Brazil; § Instituto de Física, Universidade de São Paulo, CP 66318, São Paulo, São Paulo 05315-970, Brazil

## Abstract

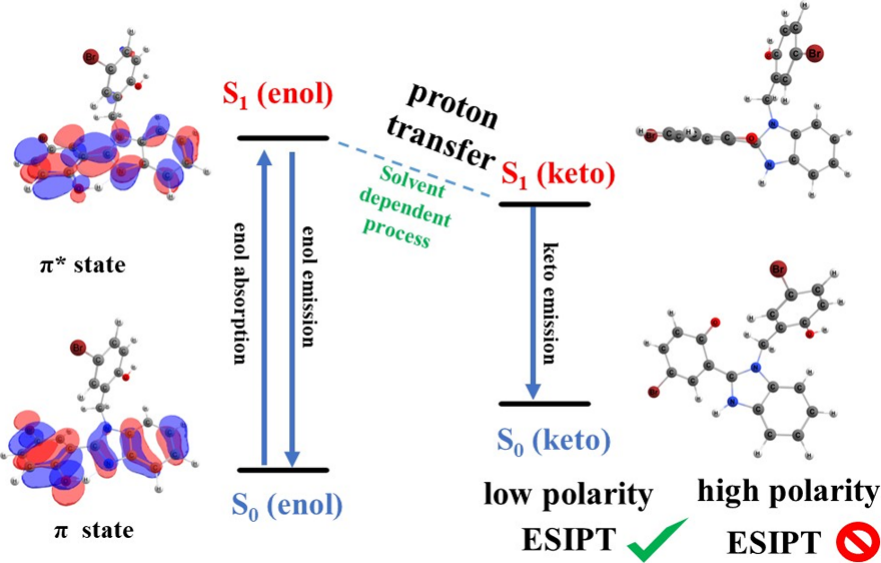

This work reports a theoretical investigation of the solvent polarity as well as the halogenation of benzimidazole derivatives during excited state intramolecular proton transfer (ESIPT). It details how the environment and halogen substitution may contribute to the efficiency of ESIPT upon keto–enol tautomerism and exploits this effect to design fluorescence sensing. For this purpose, we first examine the conformational equilibrium of benzimidazole derivatives containing different halogen atoms, which results in intramolecular proton transfer, using density-functional theory (DFT) combined with the polarizable continuum model (PCM). Then we evaluate the fluorescence of the benzimidazole derivatives in different dielectric constants within time-dependent DFT (TD-DFT) approaches. Our results quantitatively allow the determination of large Stokes shifts in nonpolar solvents around 100 nm. These theoretical results are in agreement with experimental solvatochromism studies of benzimidazoles. The effect of halogenation, with fluorine, chlorine, and bromine, is less important than solvent polarization when ESIPT takes place. Thus, halogenation can be properly chosen depending on the interest of the synthesis of benzimidazole-based turn-on fluorescence in appropriate solvents.

## 1 Introduction

Intramolecular proton transfer (IPT) is an ultrafast reaction of importance in several types of organic and biological molecules mainly because of its practical applications.^1−8^ In general, the IPT mechanism appears to be simple, although it cannot always be easily observed spectroscopically.^9^ However, IPT may occur in excited states (ES) leading to the so-called ESIPT process. This phenomenon naturally occurs in some aromatic molecules that exhibit structural flexibility to deliver a proton upon electronic excitation and is useful to design turn-on fluorescent probes;^10−18^ for example, when involving molecules that contain an enolic (or phenolic) group able to form an intramolecular H-bond with a nearby heteroatom.^19,20^ Thus, during the keto–enol tautomerism, ESIPT may be accompanied by fluorescence spectroscopy.^21−24^

To understand external/internal influences on the ESIPT process, we first consider a molecule, capable of forming an intramolecular H-bond, in its singlet electronic ground state, S_0_, in a specific solvent at room temperature. Then, this molecule is excited, for example, to the electronic singlet state S_1_, which enhances the acidity of the proton involved in the intramolecular H-bond. This allows, after a vibrational relaxation to a lower vibrational state within the S_1_ electronic state, for the process to be followed via fluorescence.^25,26^ Usually, this process occurs on a time scale of femtoseconds^27,28^ and its spectral band is quite sensitive to the environment (an external effect)^29−31^ as well as to a substituent (an internal effect) in the chromophore. The analysis of such effects can help to design efficient ESIPT processes.

A current application of ESIPT in organic compounds considers molecules containing an imidazole group;^32^ thanks to their superlative optical properties, giving rise to laser dyes.^33,34^ However, unlike the behavior of organic molecules in solution that provides a high fluorescence quantum yield,^35^ in the solid state, the fluorescence weakens or even quenches,^36,37^ limiting the applications of ESIPT in this condition. An important group of organic molecules with which we can investigate the effects of solvent, as well as substituents^38−41^ on ESIPT, is the benzimidazole derivatives.^15,42,43,44^ Experimentally, it has been observed that a bathochromic shift occurs in the fluorescence band of certain benzimidazole derivatives with increasing solvent polarity. However, high-polarity solvents also can inhibit ESIPT. Furthermore, protic solvents can prevent intramolecular H-bond formation, avoiding ESIPT.^45^

In the present work, we have investigated the role of solvent polarity as well as halogenation on the ESIPT process of benzimidazole derivatives and their implications for designing optimized molecules for fluorescence sensing. We employ DFT combined with the solvent-implicit PCM scheme to simulate the different dielectric constants of a set of solvents and calculate their effect on the UV–vis absorption and emission spectra via TD-DFT approaches. First, we determine the optimized S_0_ states of the benzimidazole derivatives, within DFT methods for different solvent models (varying the dielectric constant from *n*-hexane to water), considering their enolic forms. Second, we model the tautomerism processes in the excited S_1_ states within TD-DFT. Finally, after vibrational relaxation, we examine ESIPT by simulating the photophysical cycle with fluorescence.

## 2 Computational Methods

**Figure 1:**
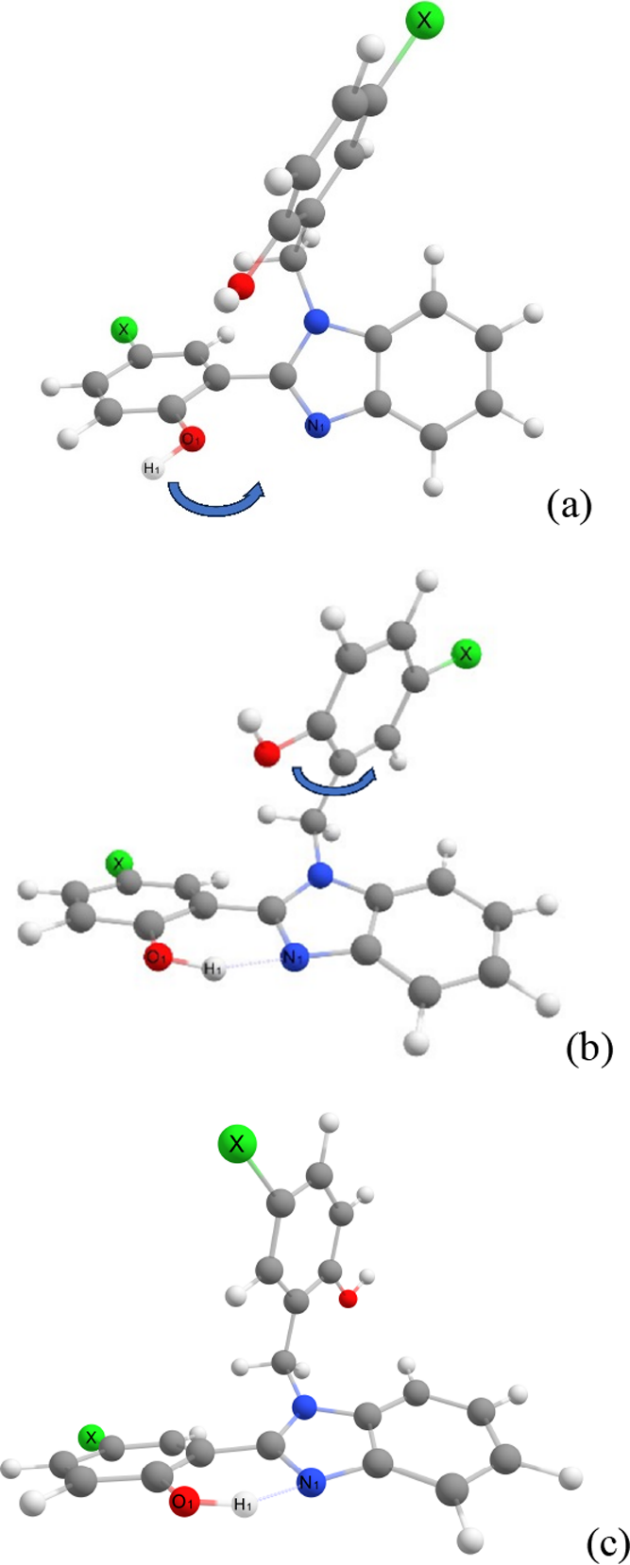
Schematic view of the conformational equilibrium for the benzimidazole derivatives in the ground (S_0_) state: (a) Conformer I, (b) Conformer II, and (c) Conformer III. The main motions are indicated with blue arrows.

We obtain the ground state optimized geometries of the benzimidazole derivatives I, II, and III (Figure 1) within DFT using the B3LYP^46−48^/6-31G(d,p) level of approximation, where X = H, F, Cl, and Br, which is a reasonable method for describing these sizes of systems.^15^ After performing the conformational analysis of the enolic forms in the different solvent environments (*n*-hexane, 1,4-dioxane, ethanol, acetonitrile, and water) within PCM,^49^ we calculate the UV–vis absorption properties of the conformers with TD-DFT^50^ using Coulomb-attenuating method CAM-B3LYP^51^/6-31G(d,p) in all these solvents. All solvents considered here exhibit increasing polarity indices that increase with their dielectric constants so that low (high) polarity coincides with their low (high) dielectric constant. The optimized structures were checked by performing frequency calculations within the harmonic approximation. We perform all calculations and analyses using the Gaussian 09^52^ suite of programs.

## 3 Results and Discussion

### 3.1 Conformational Analysis of the Benzimidazole Derivatives in the Ground State

**Figure 2:**
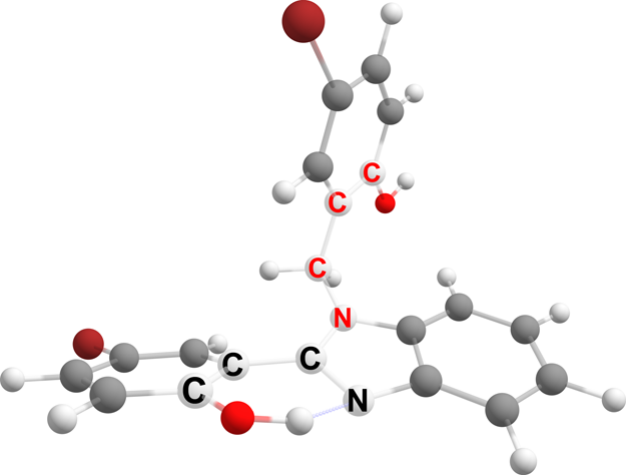
Illustration of the dihedral angles (C–C–C–N): = ϕ (labels in black) and (N–C–C–C): = ψ (labels in red) in structure III.

In the following, we discuss the molecular properties and structural features for analyzing the occurrence of tautomeric equilibrium in the excited state for solvents with increasing polarity. Figure 1 displays the optimized geometries of benzimidazole derivative I–III in their S_0_ states. After obtaining enol tautomer III, the possible keto tautomer is obtained in its S_1_ excited state in different solvents. We will discuss the excited state structures later in this paper. The atom indicated by X represents hydrogen or halogens (F, Cl, or Br) and we provide the Cartesian coordinates for each structure in the Supporting Information. The relaxation process in S_0_ consists of two steps: (a) formation of an intramolecular H-bond, O–H···N, by rotating the O–H group (cf. indicated motion in Figure 1a); and (b) rotation of the perpendicular aromatic ring to the chromophore plane (cf. indicated motion in Figure 1b). The relaxed dihedral angles ϕ and ψ after (a) and (b), illustrated in Figure 2, are reported in Table 1, together with other structural parameters for the different substituents and solvents.

**Table 1:** Calculated O_1_–H_1_ and H_1_–N_1_ Bond Lengths (in Å), Dihedral Angles ϕ and ψ (in Degrees), Dipole Moments (μ), and Dipole Moment Variations (in D) for Structures II and III in S_0_ at the B3LYP/6-31G(d,p) Level of Theory

		H		F		Cl		Br	
solvent		II	III	II	III	II	III	II	III
*n*-hexane	O_1_–H_1_	0.995	0.995	0.994	0.994	0.996	0.996	0.996	0.996
	H_1_–N_1_	1.731	1.730	1.738	1.734	1.727	1.728	1.726	1.727
	ϕ	26.9	27.0	26.8	26.4	26.9	26.5	26.7	26.4
	ψ	–63.1	116.5	–63.2	117.8	–64.1	117.4	–62.7	117.2
	μ	5.02	6.02	3.51	6.22	3.29	6.50	3.37	6.43
	Δμ	1.00		2.71		3.21		3.06	
1,4-dioxane	O_1_–H_1_	0.995	0.995	0.994	0.994	0.996	0.996	0.996	0.996
	H_1_–N_1_	1.730	1.729	1.736	1.733	1.726	1.727	1.724	1.725
	ϕ	26.9	27.3	26.8	26.4	26.9	26.5	26.7	26.4
	ψ	–63.2	116.5	–63.2	117.7	–64.1	117.4	–62.7	117.2
	μ	5.13	6.15	3.57	6.36	3.33	6.64	3.41	6.58
	Δμ	1.03		2.78		3.32		3.16	
ethanol	O_1_–H_1_	0.996	0.996	0.995	0.995	0.997	0.997	0.997	0.997
	H_1_–N_1_	1.722	1.724	1.727	1.726	1.717	1.720	1.716	1.718
	ϕ	26.9	27.3	26.8	26.7	26.9	26.9	26.8	26.9
	ψ	–63.6	116.0	–63.6	117.0	–64.2	116.8	–62.7	116.7
	μ	5.96	7.16	4.05	7.36	3.57	7.72	3.67	7.67
	Δμ	1.20		3.31		4.15		4.00	
acetonitrile	O_1_–H_1_	0.996	0.996	0.995	0.995	0.997	0.997	0.998	0.997
	H_1_–N_1_	1.722	1.724	1.726	1.726	1.717	1.720	1.715	1.718
	ϕ	26.9	27.3	26.8	26.7	26.9	26.9	26.8	26.9
	ψ	–63.6	116.0	–63.7	116.9	–64.2	116.8	–62.7	116.8
	μ	6.00	7.20	4.07	7.40	3.58	7.76	3.68	7.72
	Δμ	1.20		3.33		4.18		4.04	
water	O_1_–H_1_	0.996	0.996	0.995	0.996	0.997	0.998	0.998	0.997
	H_1_–N_1_	1.722	1.724	1.726	1.726	1.717	1.715	1.715	1.718
	ϕ	26.9	27.4	26.8	26.8	26.9	27.0	26.8	26.9
	ψ	–63.6	116.0	–63.7	116.9	–64.2	116.8	–62.8	116.7
	μ	6.04	7.26	4.09	7.46	3.60	7.82	3.69	7.78
	Δμ	1.22		3.37		4.23		4.09	

**Figure 3:**
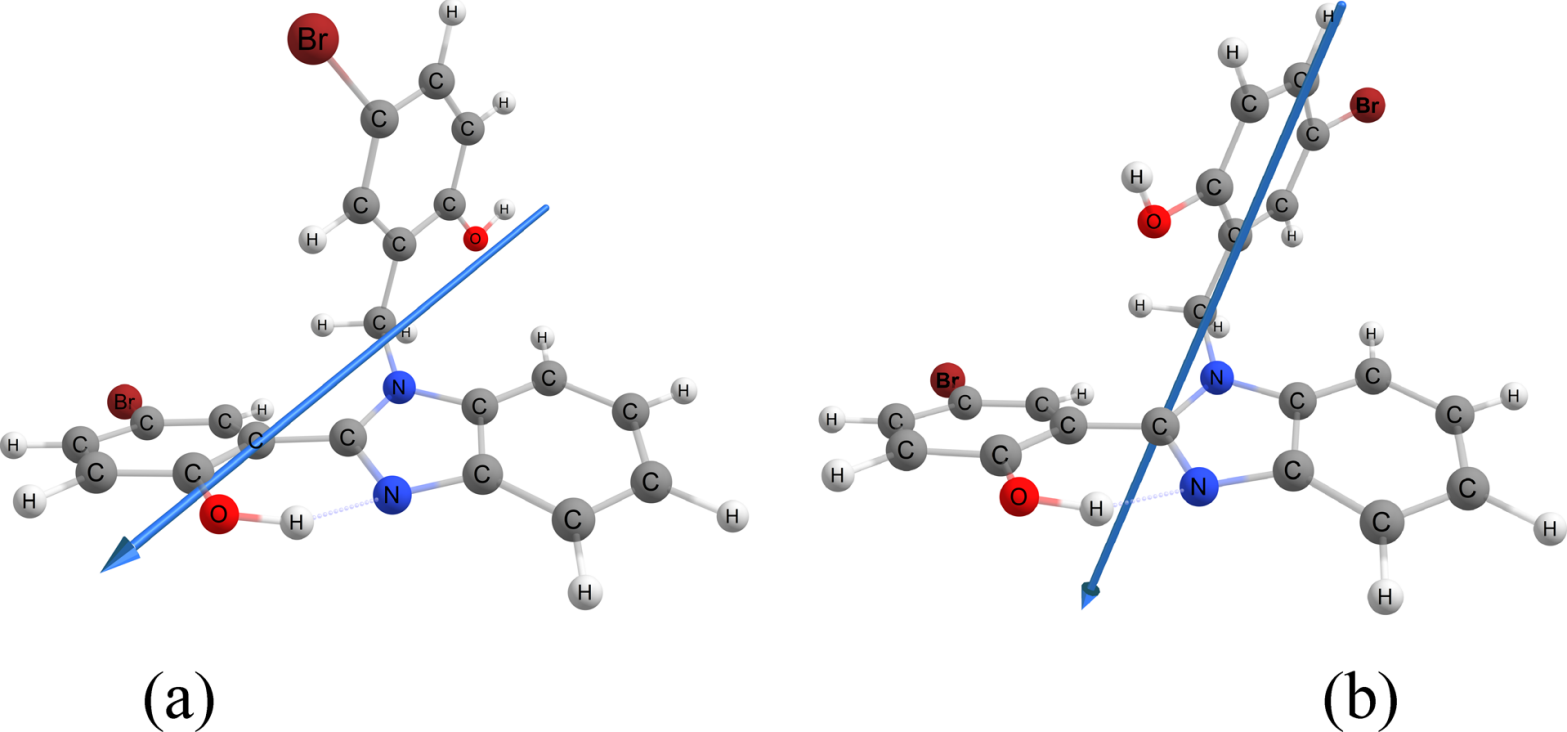
Relative orientation of the dipole moments in enolic (E) conformers II (a) and III (b) in S_0_ for X = Br in *n*-hexane.

As summarized in Table 1, these geometric parameters are not very sensitive either to the type of halogen or to the type of solvent. In general, the H_1_–N_1_ distance in III is around 1.73 Å in low-polarity solvents and 1.72 Å in high-polarity solvents, considering X = H, Cl, and Br, with the exception of X = F that maintains the H_1_–N_1_ distance around 1.73 Å in all considered solvents. Similarly, the equilibrium dihedral angle (ψ) in III is almost constant under these conditions. The great changes in the structures of these conformers, however, are observed in the dipole moments of III and dipole moment variations between structures II and III. As reported in Table 1, there is a small variation Δμ for X = H of 1.00 D in *n*-hexane to 1.22 D in water. Interestingly, this variation becomes maximal for X = Cl (Br), that is, Δμ = 3.21 D (Δμ = 3.06 D) in *n*-hexane and Δμ = 4.23 D (Δμ = 4.09 D) in water. These changes indicate that a relatively higher dipole moment variation, as a function of the dielectric constant, prevents ESIPT, since III is the conformer in which IPT may occur. The relative orientation of the dipole moments of II and III is illustrated in Figure 3, which are calculated as 3.37 and 6.43 D, respectively, for X = Br in *n*-hexane. If the difference between these two dipole moments increases significantly (approximately 4 D within these calculations), the O–H···N proton transfer is electrostatically hindered.

Another property that may be related to ESIPT is the shift in the vibrational stretching mode of the O–H group forming a H-bond with N at the imidazole ring (cf. Figure 2). In order to have a first estimate of the medium impact on this mode, we report in Table 2 the calculated redshift associated with the vibrational stretching upon transferring the molecules to a specific solvent (i.e., from vacuum to a specific dielectric constant), considering the different types of halogens. Considering the purely hydrogenated systems, the redshift increases toward higher polarity. For example, we determine an amount of 18.3 cm^–1^ in *n*-hexane to 43.7 cm^–1^ in water in this derivative with X = H. At low polarity, these redshifts slightly increase in X = H and Br, being maximal in X = Cl with 27.4 cm^–1^, from vacuum to 1,4-dioxane. From moderate to high polarity, these redshifts increase in X = H and Br, reaching the maximum value of 58.9 cm^–1^ for X = Br in water.

**Table 2:** Calculated Shifts of the O–H Vibrational Stretching (in cm^–1^) of Conformer III, Related to the Corresponding Vacuum Equilibrium Structure at the B3LYP/6-31G(d,p) Level of Theory

	H		F		Cl		Br	
solvent	ν_O–H_	Δν	ν_O–H_	Δν	ν_O–H_	Δν	ν_O–H_	Δν
*n*-hexane	3230.7	–18.3	3246.4	–20.1	3219.8	–23.1	3217.9	–22.6
1,4-dioxane	3227.9	–21.1	3242.1	–24.4	3215.4	–27.4	3213.5	–27.0
ethanol	3205.8	–43.2	3216.6	–50.0	3187.7	–55.2	3183.4	–57.1
acetonitrile	3205.9	–43.1	3215.6	–50.9	3187.2	–55.6	3182.8	–57.7
water	3205.3	–43.7	3214.6	–52.0	3187.0	–55.9	3181.6	–58.9

To evaluate the energetics of conformers I, II, and III, we have considered steps (a) and (b) described in Figure 1. As reported in Table 3, the H-bond formation leading to conformer II accounts for a great amount of energy (∼10 kcal/mol) in low-polarity solvents. This relative energy decreases to ∼7 kcal/mol from ethanol to water, also indicating that protic solvents could compete with the intramolecular H-bond. Consequently, the ESIPT could be hampered by a high-polarity medium. In addition, in low-polarity solvents, we find that the relative energy between structures III and II is higher for X = H and F (0.6 and 0.5 kcal/mol, respectively) and tends to decrease for X = Cl and Br (about 0.2 kcal/mol). On the other hand, this difference is lower (0.2–0.4 kcal/mol) in more polar solvents, independently of the type of the substituted halogen.

**Table 3:** Calculated Energy Difference (in kcal/mol) for the O–H Bond Torsion (I → II) and Conformational Equilibrium (II → III) for Different Substituents and Solvents, at the B3LYP/6-31G(d,p) Level of Theory (cf. Figure 1)

	H		F		Cl		Br	
solvent	Δ*E*_I→II_	Δ*E*_II→III_	Δ*E*_I→II_	Δ*E*_II→III_	Δ*E*_I→II_	Δ*E*_II→III_	Δ*E*_I→II_	Δ*E*_II→III_
*n*-hexane	–9.84	–0.58	–9.70	–0.53	–9.84	–0.21	–9.88	–0.21
1,4-dioxane	–9.56	–0.55	–9.41	–0.51	–9.53	–0.21	–9.58	–0.20
ethanol	–7.07	–0.29	–6.91	–0.37	–6.92	–0.21	–6.96	–0.21
acetonitrile	–6.95	–0.28	–6.95	–0.37	–6.80	–0.23	–6.83	–0.21
water	–6.80	–0.26	–6.63	–0.36	–6.67	–0.21	–6.67	–0.21

### 3.2 UV Absorption Bands of the Enol Conformers from Vertical Excitations

First, we consider the vertical excitation from S_0_ to S_1_ for the three conformers displayed in Figure 1, using the B3LYP/6-31G(d,p) scheme within TD-DFT. In Table 4, we report the absorption wavelengths (λ) and oscillator strength (*f*) for these structures with X = H, F, Cl, and Br in different solvents. From structure I, we examine the effect of H-bonding during the photoabsorption in structure II. For example, before the intramolecular H-bond formation, the absorption of I takes place in the limit between the UVC and UVB regions (∼280 nm). For X = H, the absorption band varies from 277.9 nm (in *n*-hexane) to 280.8 nm (in water), increasing by ∼10 nm for X = F. Upon halogenation with X = Cl or Br, the absorption band becomes constant at ∼288 nm for all solvents. There is, however, a large redshift in the absorption band upon H-bond formation in II. This occurs now in the UVA region (above 315 nm), with the values being more sensitive to the solvent polarity. For example, considering X = F, the absorption band reads 324–325 nm in low polarity solvents and 329–330 nm in solvents with higher polarities.

**Table 4:** Calculated Absorption Wavelengths (λ in nm) and Oscillator Strengths (*f*) of Conformers I, II, and III with X = H, F, Cl, and Br, in Different Solvents, with TD-B3LYP/6-31G(d,p)

I								
	H		F		Cl		Br	
solvent	λ	*f*	λ	*f*	λ	*f*	λ	*f*
*n*-hexane	277.9	0.384	287.6	0.357	288.4	0.349	288.4	0.343
1,4-dioxane	278.0	0.384	287.7	0.357	288.2	0.372	288.2	0.364
ethanol	280.7	0.588	288.7	0.524	287.7	0.528	288.7	0.512
acetonitrile	280.8	0.594	288.9	0.529	287.7	0.532	288.8	0.518
water	280.8	0.594	289.0	0.530	287.6	0.538	288.8	0.523

II								
	H		F		Cl		Br	
Solvent	λ	*f*	λ	*f*	λ	*f*	λ	*f*
*n*-hexane	312.2	0.481	324.3	0.438	321.6	0.436	323.1	0.418
1,4-dioxane	312.8	0.509	324.9	0.464	322.1	0.462	323.5	0.444
ethanol	317.4	0.715	329.1	0.647	326.0	0.656	327.2	0.635
acetonitrile	317.6	0.724	329.3	0.654	326.2	0.664	327.4	0.643
water	317.8	0.734	329.5	0.664	326.4	0.674	327.6	0.653

III								
	H		F		Cl		Br	
solvent	λ	*f*	λ	*f*	λ	*f*	λ	*f*
*n*-hexane	313.7	0.479	326.4	0.440	323.7	0.431	325.4	0.407
1,4-dioxane	314.3	0.506	326.9	0.465	324.2	0.458	325.8	0.433
ethanol	318.6	0.710	330.8	0.647	327.4	0.655	328.9	0.629
acetonitrile	318.8	0.719	330.9	0.654	327.6	0.663	329.0	0.637
water	319.9	0.729	331.1	0.663	327.7	0.673	329.2	0.647

Upon dihedral relaxation leading to halogenated structures III, which correspond to an experimental structure in the case of X = Br^45^ and are candidates that exhibit ESIPT, the UV absorption trend remains in the same region, although reading ∼2 nm above the absorption profile of structures II. As expected at this level of approximation, because of the lack of Coulomb attenuation during the electronic excitation calculations, the absorption is overestimated, mainly in higher dielectric constants. This result, however, does not invalidate the preliminary analysis of the role of the solvent and impact of halogenation on these benzimidazole derivatives. Moreover, as we discuss in the following, the inclusion of Coulomb attenuation into B3LYP can resolve this issue.

**Figure 4:**
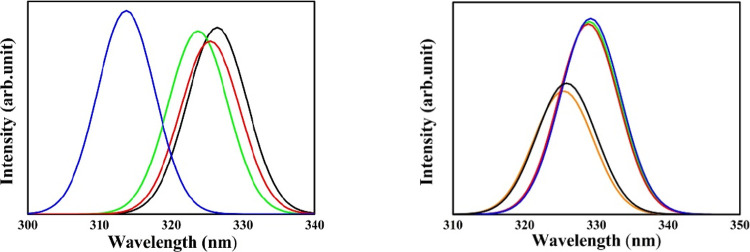
Calculated absorption spectra of III. Left panel: effect of the different substituents, where color lines refer to H (blue), F (black), Cl (green), and Br (red) in *n*-hexane. Right panel: solvent effect where color lines refer to *n*-hexane (orange), 1,4-dioxane (black), ethanol (red), acetonitrile (green), and water (blue) for X = Br.

In Figure 4, we display the separate impact of both substituent and solvent polarity for structure III. Now, it is clear to notice a significant redshift (max 12.7 nm for X = F from the hydrogenated structure) upon halogenation of the benzimidazole derivatives, as indicated in the left panel for the structures in *n*-hexane. In the right panel, the solvent effect is also clearly observed with an appreciable redshift to high-polarity solvents (3.8 nm for X = Br from *n*-hexane to water). Furthermore, we notice an increase in the absorption bands for this structure in solvents with high dielectric constants, as indicated by the oscillator strengths in Table 4. These are around 0.4 in low polarity and 0.6 in high polarity for X = Br. In turn, the absorption intensities of the purely hydrogenated benzimidazole derivative are higher (*f* = 0.5–0.7) than the halogenated species.

**Figure 5:**
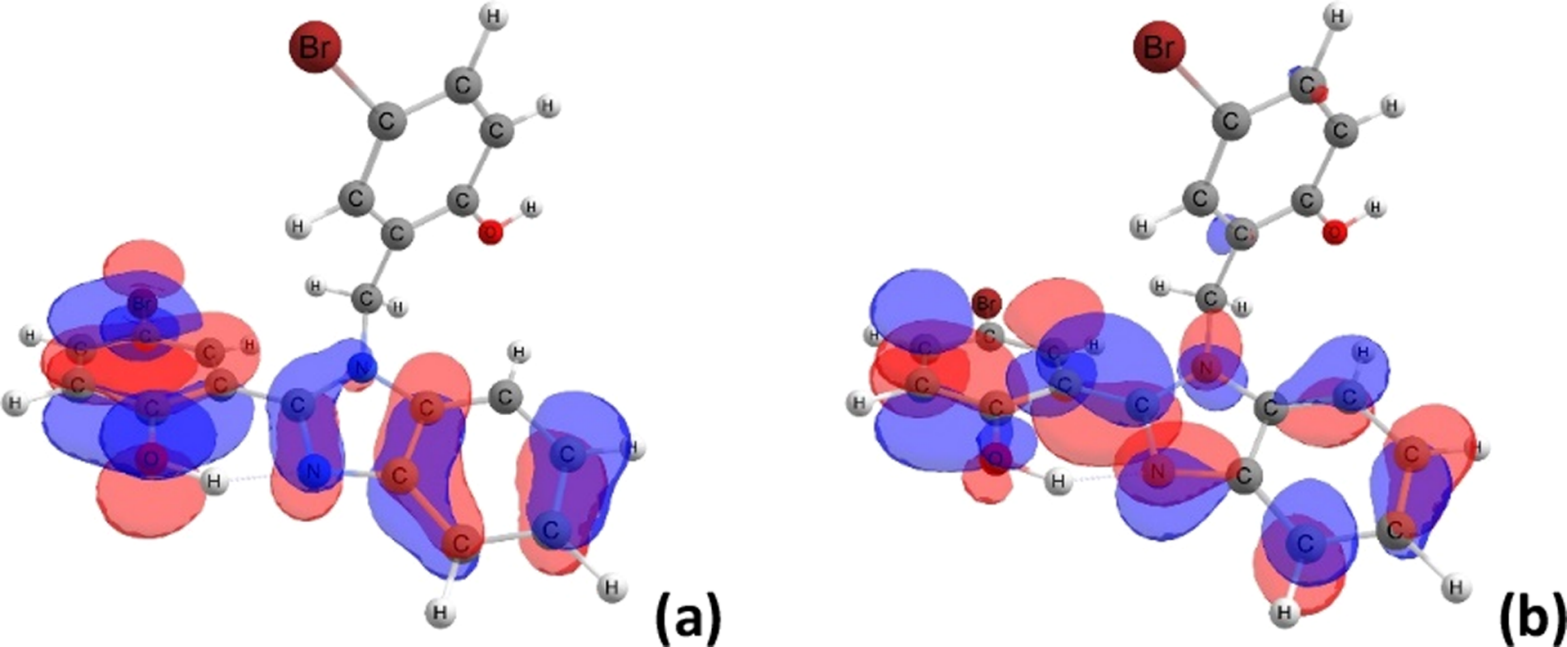
Frontier molecular orbitals (isosurface contour value: 0.03): HOMO (a) and LUMO (b) of structure III within TD-DFT [B3LYP/6-31G(d,p)] in *n*-hexane indicate the chromophore in these molecules.

As a complement to this analysis, we display the frontier molecular orbitals involved in the vertical excitations of III (with X = Br) from S_0_ to S_1_ in Figure 5. As displayed within TD-DFT, the HOMO and LUMO are delocalized on the chromophore, exhibiting π- and π*-type characters for all benzimidazole derivatives. We notice that the halogen atom at the chromophore contributes to the HOMO but is completely devoid of charge in the LUMO density. Additionally, we report the Mulliken charges in the O–H···N bond in both ground and excited states to estimate the charge transfer (CT) during the π → π* excitation (cf. Table S2). Atomic charges are not true observables, and the results used here, of course, are for qualitative analysis and trends. We notice, in all cases, the same CT trend from S_0_ to S_1_, where O_1_ loses and N_1_ gains electron charge, even in solvents with low polarities, such as *n*-hexane and 1,4-dioxane.

At this point, it is important to mention that the results obtained for the electronic excitations can be improved by using the CAM-B3LYP/6-31G(d,p) level of approximation. Indeed, according to this calculation, the UV bands are in better agreement with the available experimental data.^42^ For the case of X = Br in *n*-hexane, the experimental value is 296.4 nm, whereas the theoretical prediction using CAM-B3LYP is 296.3 nm. A similar agreement is obtained in 1,4-dioxane, for which the experimental band reads 295.6 nm, and the calculated value is 296.7 nm. The theoretical result is somewhat less accurate in the case of more polar solvents, as noticed for the case of acetonitrile. Now, the experimental band is 288.5 nm, whereas the theoretical value is 299.6 nm. From these new results, we proceeded to investigate the ESIPT of these systems using CAM-B3LYP.

### 3.3 Turning on the ESIPT Process from III during the Keto–Enol Tautomerism

**Figure 6:**
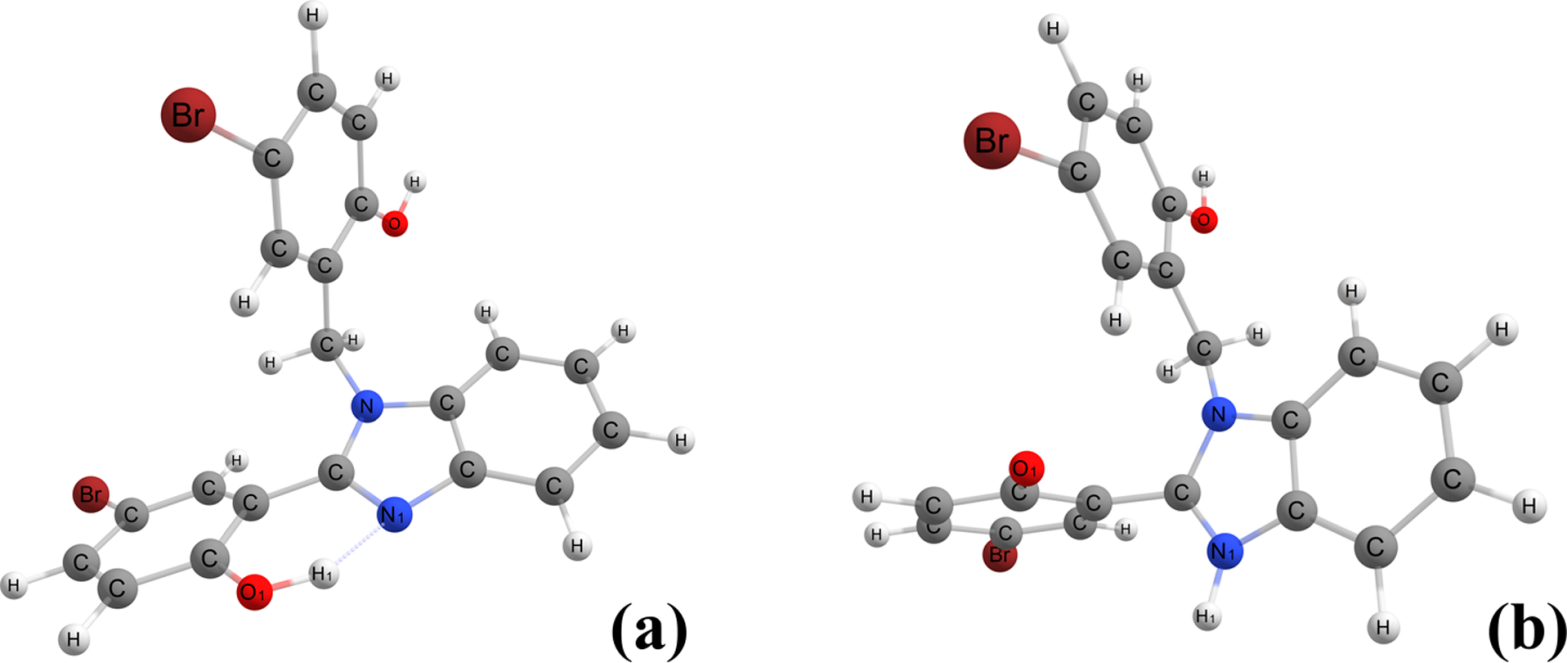
Optimized geometries of structure III (X = Br) in excited state S_1_ using B3LYP/6-31G(d,p) in *n*-hexane. Enol tautomer (a) upon the vertical excitation and keto tautomer (b) upon vibrational relaxation resulting in structure IV(K*).

To simulate ESIPT, we consider the excited states S_1_ of enol tautomers III (X = H, F, Cl, and Br) using the CAM-B3LYP scheme in the different solvents, leading to structure IV. In Figure 6, we illustrate a case where the IPT takes place, resulting in the keto tautomer IV in S_1_ (K*). In Table 5, we summarize all relaxed structures to IV, indicating the structures in which the keto–enol tautomerism actually takes place by the changes in the O_1_–H_1_ and H_1_–N_1_ distances. Additionally, we report the dipole moment variation in the excited states. Now, it is clear that IPT does occur only in low-polarity solvents, such as *n*-hexane and 1,4-dioxane, which is in agreement with experimental results for correlated benzimidazole derivatives.^45^

**Table 5:** Calculated O_1_–H_1_ and H_1_–N_1_ Bond Lengths (in Å) and Dipole Moment Variation Δμ* (in D) for III in S_0_ and Relaxed to IV in the S_1_ Excited State. Bond Lengths Calculated Using B3LYP and Δμ* Calculated with CAM-B3LYP

*n*-hexane								
	H		F		Cl		Br	
	III	IV	III	IV	III	IV	III	IV
O_1_–H_1_	0.995	3.429	0.994	3.449	0.996	3.968	0.996	3.472
H_1_–N_1_	1.730	1.006	1.734	1.006	1.728	1.009	1.727	1.006
Δμ*	3.60		4.52		7.68		5.11	

1,4-dioxane								
	H		F		Cl		Br	
	III	IV	III	IV	III	IV	III	IV
O_1_–H_1_	0.995	3.429	0.994	3.463	0.996	3.460	0.996	3.486
H_1_–N_1_	1.729	1.006	1.733	1.006	1.727	1.006	1.725	1.006
Δμ*	–1.02		–1.59		–1.64		–1.37	

ethanol								
	H		F		Cl		Br	
	III	IV	III	IV	III	IV	III	IV
O_1_–H_1_	0.996	1.035	0.995	1.062	0.997	1.048	0.997	1.050
H_1_–N_1_	1.724	1.555	1.726	1.486	1.720	1.520	1.718	1.515
Δμ*	–0.05		0.36		0.31		0.32	

acetonitrile								
	H		F		Cl		Br	
	III	IV	III	IV	III	IV	III	IV
O_1_–H_1_	0.996	1.035	0.995	1.061	0.997	1.047	0.997	1.049
H_1_–N_1_	1.724	1.556	1.726	1.490	1.720	1.523	1.718	1.517
Δμ*	–0.06		0.34		0.30		0.31	

water								
	H		F		Cl		Br	
	III	IV	III	IV	III	IV	III	IV
O_1_–H_1_	0.996	1.034	0.996	1.059	0.997	1.046	0.997	1.048
H_1_–N_1_	1.724	1.558	1.726	1.494	1.720	1.525	1.718	1.520
Δμ*	–0.96		–1.64		–0.86		–1.06	

**Figure 7:**
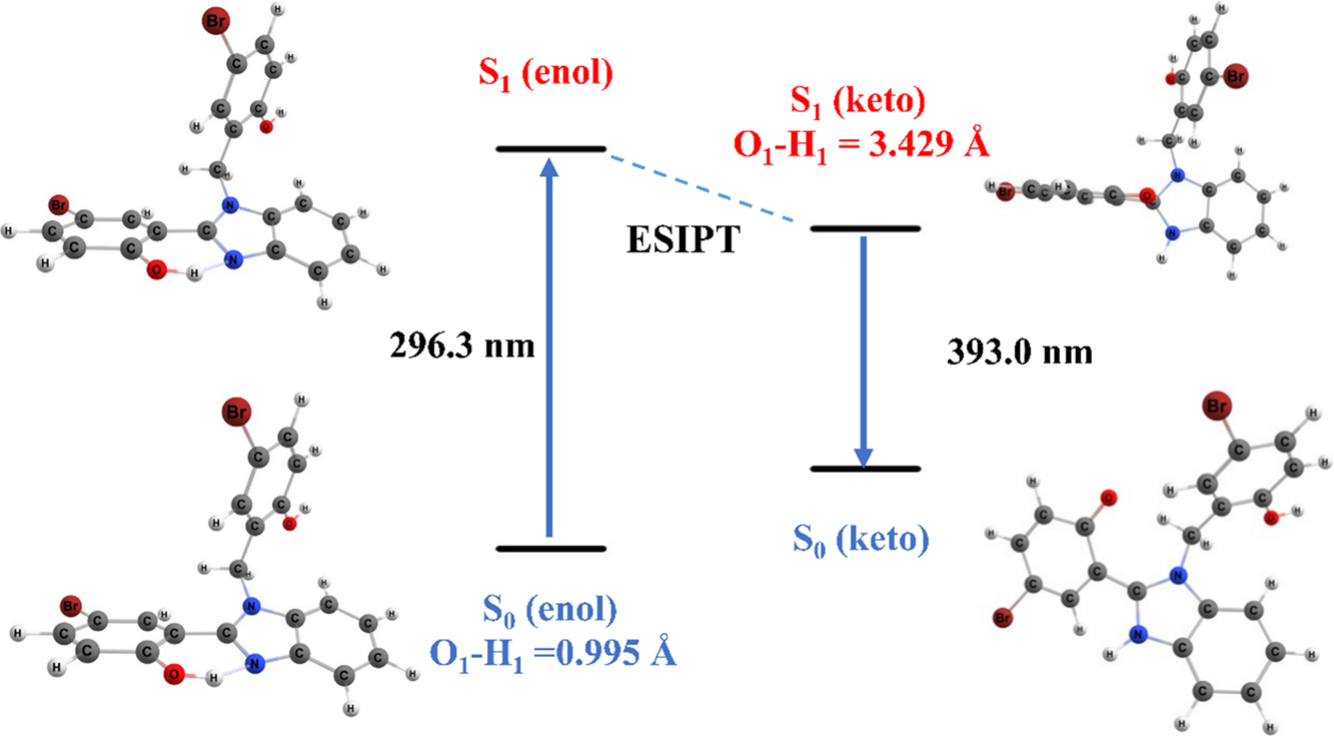
Photophysical cycle for the ESIPT process of the benzimidazole derivatives.

In high-polarity environments, such as ethanol and acetonitrile, we notice only a small increase in the O_1_–H_1_ distance (∼0.04 Å) and a large decrease in the H_1_–N_1_ distance (∼0.17 Å) from III to IV. Indeed, considering the high-polarity solvents, both structures III and IV remain as enol tautomers upon relaxation of their excited states (cf. Figure 7). The variations in these distances, however, reflect the charge redistribution in the excited states. From this analysis, the solvent with high dielectric constants do not favor ESIPT, which is in line with available experimental data for benzimidazole derivatives.^45^

Based on this investigation about the environment and substituent effects, it is now possible to investigate the fluorescence process for the benzimidazole derivatives that exhibit ESIPT. Thus, we consider (i) a vertical excitation from III (S_0_) → III (S_1_), (ii) the relaxation in the excited state from III (S_1_) → IV (S_1_), and (iii) IV (S_1_) → V (S_0_). We illustrate the general scheme in Figure 7 for the case where X = Br in *n*-hexane. The calculated emission bands and the corresponding Stokes shifts, that is, the difference between the absorbed and emitted wavelengths of a fluorescent molecule, are reported in Table 6. The smallest emission is calculated for the benzimidazole derivative with X = H (∼387.3 nm), and largest emission occurs for X = F, around 402.0 nm. For X = Cl and Br, the emission bands are 393.1 and 393.2 nm, respectively.

**Table 6:** Calculated Fluorescence Bands and Stokes Shifts (in nm) for ESIPT in Low-Polarity Solvents Using CAM-B3LYP/6-31G(d,p)

*n*-hexane				
	H	F	Cl	Br
fluorescence	387.3	402.0	393.1	393.2
stokes shift	98.7	102.8	97.3	96.9

1,4-dioxane				
	H	F	Cl	Br
fluorescence	387.4	402.3	392.9	392.9
stokes shift	98.3	102.7	96.6	96.2

**Figure 8:**
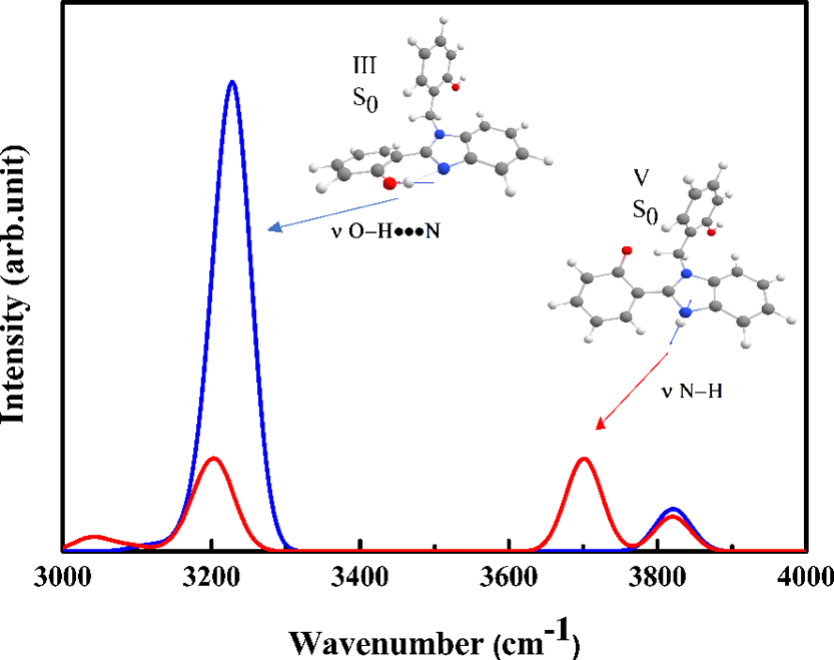
Calculated infrared spectra of III and V in the stretching region for X = H in *n*-hexane. Lines in blue and red refer to III and V tautomers in S_0_, respectively.

Considering that ESIPT is favored in low polarities, the obtained values for the fluorescence and Stokes shift, calculated using CAM-B3LYP, were found to be similar in *n*-hexane and 1,4-dioxane. For instance, in the case of X = Br, in *n*-Hexane, we observe emission at 393.2 nm with a Stokes shift of 96.9 nm, while in 1,4-dioxane, the corresponding values were 392.9 and 96.2 nm, respectively. These results suggest that in cases where ESIPT occurs both fluorescence and Stokes shift appear to be less sensitive to variations of solvent polarity. On the other hand, these properties appear to be more sensitive to the substituent. For instance, when X = F, in *n*-hexane, the calculated fluorescence and Stokes shift yields the highest values, that is, 402.0 and 102.8 nm, respectively. Considering practical applications, the choice of a suitable substituent may contribute to the reduction of self-absorption. Furthermore, an interesting approach to further investigate the ESIPT in benzimidazole derivatives is by using infrared spectroscopy (IR). We can note that the N–H stretching of tautomer V is absent in III, as realized in Figure 8.

As indicated in Table 2, the O–H stretching vibrational frequencies of conformer III decrease with increasing solvent polarity. On the contrary, considering that the tautomer V exists only in low polarity, the N–H vibrational mode is less sensitive to the medium effect. For instance, the purely hydrogenated system reads values between 3672 and 3674 cm^–1^ in low polarity for this vibrational mode. This is illustrated in Figure 8. Table 7 exhibits the calculated values for the N–H stretching mode and the corresponding differences between this mode and the O–H···N stretching mode of tautomer III for different substituents in low-polarity solvents.

**Table 7:** Calculated N–H Vibrational Stretching Modes (in cm^–1^) for Tautomer V in Different Solvents and Shifts (in cm^–1^) Related to O–H Stretching Vibrational Mode Using B3LYP/6-31G(d,p)

	H		F		Cl		Br	
solvent	ν_N–H_	Δν _(to O–H)_	ν_N–H_	Δν _(to O–H)_	ν_N–H_	Δν _(to O–H)_	ν_N–H_	Δν _(to O–H)_
*n*-hexane	3674.6	443.9	3673.8	427.4	3669.7	449.9	3670.1	452.2
1,4-dioxane	3672.8	444.9	3672.2	430.1	3668.1	452.7	3668.3	454.8

We notice now that the N–H stretching modes appear to be more sensitive to halogenation than the medium. For instance, in *n*-hexane, it reads 3673.8 and 3670.1 cm^–1^ for X = F and Br, respectively. On the other hand, their differences with respect to the O–H···N stretching of tautomer III are only slightly sensitive to halogenation. In the case of X = Br, the highest values are 452.2 and 454.8 cm^–1^ for *n*-hexane and 1,4-dioxane, respectively; while for the purely hydrogenated system, the corresponding values are 443.9 and 444.9 cm^–1^. In this sense, this vibrational difference can be used to follow the ESIPT mechanism in benzimidazole derivatives.

## 4 Conclusions

We theoretically investigated the ESIPT process of some benzimidazole derivatives using TD-DFT approaches. Our examination focused on understanding the influence of solvent polarity and the substitution of different halogen atoms in facilitating ESIPT, with implications for the design of turn-on fluorescent probes for sensing.

Initially, we assessed the conformational equilibrium and the role of the dipole moment variation of benzimidazole derivatives in the ground state across different solvent polarities and different halogenations. Our findings highlight that slight variation in dipole moment, that is, around 1.0 D, in low polarity environments under different halogenations is sufficient to favor ESIPT. However, when the same magnitude of variation occurs in higher polarities, the process is not achieved. This dipole moment variation proves to be of relevance to ESIPT, particularly for enolic conformers, which are amenable to intermolecular proton transfer.

Another ground-state property influencing the ESIPT is the redshift of the O–H stretching band upon forming a hydrogen bond with the N atom of the imidazole ring. Our results also reveal a significant variation in the redshift from vacuum to high-polarity environments, suggesting a potential correlation with ESIPT. For example, in the case of Br^–^ benzimidazole derivatives in low-polarity solvents, such as *n*-hexane and 1,4-dioxane, the calculated redshifts of the OH stretching were of 22.6 and 27.0 cm^–1^, respectively, which increased dramatically to 57.1 cm^–1^ in ethanol, indicating that higher-polarity solvents can inhibit ESIPT.

Regarding halogen substitution, its effect is found to exert a subtle influence on ESIPT, providing a valuable perspective in the synthesis of benzimidazole derivatives for turn-on fluorescence probes. These theoretical results are in line with spectral studies and solvatochromism of some benzimidazole derivatives and suggest that ESIPT is facilitated in very low-polarity solvents such as *n*-hexane and 1,4-dioxane. Conversely, our study indicates that ESIPT should not take place in high-polarity environments, such as ethanol, acetonitrile, and water. In this sense, this theoretical study provides useful information for the synthesis of benzimidazole-based turn-on fluorescence probes.

## Supporting Information Available

Cartesian coordinates of the optimized structures at the B3LYP/6-31G(d,p) level of theory and Mulliken population analysis of atomic charges in the O1–H1···N1 bond of conformers III in S0 and S1 states for different substituents and solvents (PDF)
