# Supplementary material for: Influence of Solvents and Halogenation on ESIPT of Benzimidazole Derivatives for Designing Turn-on Fluorescence Probes
Source: ACS Omega. 2024 May 10;9(20):22102–11. doi: 10.1021/acsomega.4c00488 (PMC11112562; doi:10.1021/acsomega.4c00488)
Supplement: Supplementary file 1 — ao4c00488_si_001.pdf [file ao4c00488_si_001.pdf]

Supporting Information

Influence of solvents and halogenation on ESIPT of benzimidazole derivatives for designing turn-on fluorescence probes

Murillo H. Queiroz[1], Tiago V. Alves[1], Roberto Rivelino*[2], Sylvio Canuto[3]

[1]Departamento de Físico-Química, Instituto de Química, Universidade Federal da Bahia - Rua Barão de Jeremoabo, 147, 40170-115, Salvador, Bahia, Brazil

[2]Instituto de Física, Universidade Federal da Bahia, 40210-340 Salvador, Bahia, Brazil

[3]Instituto de Física, Universidade de São Paulo, CP 66318, 05315-970 São Paulo, SP, Brazil

Table S1. Cartesian Coordinates of the Optimized Geometries of the Different Structures at B3LYP/6-31G(d,p) Level of Theory in Vacuum

H							
I				II			
6	2.547972000	1.646037000	0.723070000	6	-2.309947000	1.710688000	-0.892507000
6	1.190863000	1.543847000	0.402491000	6	-0.968129000	1.473810000	-0.580743000
6	0.519917000	2.534354000	-0.353608000	6	-0.185818000	2.427627000	0.107213000
6	1.215485000	3.663964000	-0.803874000	6	-0.740820000	3.649383000	0.506293000
6	2.565543000	3.771971000	-0.486977000	6	-2.076542000	3.886790000	0.200640000
6	3.220773000	2.776869000	0.265785000	6	-2.847542000	2.931155000	-0.489518000
7	0.224030000	0.592761000	0.695445000	7	-0.120138000	0.396423000	-0.828889000
7	-0.812240000	2.195524000	-0.515084000	7	1.098990000	1.945789000	0.258346000
6	-0.954720000	1.047392000	0.111097000	6	1.118645000	0.747473000	-0.313789000
6	-2.250526000	0.350559000	0.270411000	6	2.350098000	-0.047277000	-0.355707000
6	-2.991540000	-0.087314000	-0.845802000	6	3.320343000	0.164062000	0.665599000
6	-4.246248000	-0.679905000	-0.664336000	6	4.480046000	-0.624295000	0.691165000
6	-4.771696000	-0.848633000	0.615344000	6	4.721890000	-1.562392000	-0.304825000
6	-4.047980000	-0.428264000	1.730725000	6	3.816991000	-1.723022000	-1.359524000
6	-2.800928000	0.168037000	1.547021000	6	2.650312000	-0.967147000	-1.378102000
8	-2.445555000	0.063284000	-2.085458000	8	3.176640000	1.100528000	1.622198000
1	-3.101652000	-0.191169000	-2.748367000	1	2.416650000	1.681952000	1.349617000
1	-4.452567000	-0.550076000	2.730120000	1	4.024177000	-2.421352000	-2.163643000
6	0.459621000	-0.687389000	1.369414000	6	-0.568950000	-0.934926000	-1.266693000
6	1.419195000	-1.608200000	0.641794000	6	-1.719760000	-1.503708000	-0.462948000
6	1.088614000	-2.119708000	-0.626680000	6	-1.562044000	-1.780811000	0.907633000
6	1.970431000	-2.965040000	-1.305087000	6	-2.609844000	-2.341091000	1.643381000
6	3.184005000	-3.322522000	-0.716596000	6	-3.818323000	-2.644675000	1.016280000
6	3.519369000	-2.843509000	0.549057000	6	-3.986806000	-2.392960000	-0.344796000
6	2.634591000	-1.990875000	1.213178000	6	-2.936761000	-1.824878000	-1.068686000
1	0.845541000	-0.487759000	2.375145000	1	-0.849858000	-0.888785000	-2.324471000
1	-0.515025000	-1.166310000	1.479869000	1	0.289340000	-1.599297000	-1.178068000
1	4.459338000	-3.124124000	1.012985000	1	-4.923120000	-2.632710000	-0.837772000
8	-0.126250000	-1.761858000	-1.130837000	8	-0.349471000	-1.487696000	1.460286000
1	-0.206742000	-2.040442000	-2.052094000	1	-0.353400000	-1.712742000	2.400359000
1	3.063557000	0.881406000	1.292707000	1	-2.915776000	0.981235000	-1.415847000
1	0.702028000	4.426237000	-1.381282000	1	-0.136245000	4.381184000	1.032154000
1	3.128725000	4.637281000	-0.824071000	1	-2.535468000	4.825667000	0.495343000
1	4.276283000	2.892298000	0.494186000	1	-3.887016000	3.149509000	-0.714859000
1	-4.807747000	-1.014887000	-1.533939000	1	5.188638000	-0.456975000	1.495562000
1	-5.747513000	-1.309762000	0.735361000	1	5.633100000	-2.152804000	-0.273890000
1	-2.243046000	0.525307000	2.407850000	1	1.976226000	-1.064027000	-2.222073000
1	1.700435000	-3.346583000	-2.287019000	1	-2.473709000	-2.544176000	2.703071000
1	3.862516000	-3.980693000	-1.251104000	1	-4.625141000	-3.081574000	1.596951000
1	2.891350000	-1.610158000	2.198931000	1	-3.059944000	-1.626083000	-2.130718000
III				IV			
6	-2.264702000	1.782276000	-0.752643000	6	-2.609271000	1.609569000	-0.265913000
6	-0.914506000	1.541302000	-0.486212000	6	-1.216384000	1.648370000	-0.303108000
6	-0.106875000	2.470285000	0.203786000	6	-0.507563000	2.832155000	0.016242000
6	-0.644737000	3.685065000	0.646360000	6	-1.175002000	3.998120000	0.387382000
6	-1.987747000	3.932497000	0.383090000	6	-2.581317000	3.951885000	0.431804000
6	-2.783774000	2.995534000	-0.305800000	6	-3.280163000	2.789977000	0.109865000
7	-0.086194000	0.462279000	-0.783756000	7	-0.283951000	0.684452000	-0.651056000
7	1.175334000	1.969338000	0.314199000	7	0.827350000	2.554340000	-0.176898000
6	1.168318000	0.783068000	-0.285381000	6	1.006362000	1.249523000	-0.649309000
6	2.386462000	-0.032489000	-0.356589000	6	2.256635000	0.475069000	-0.372471000
6	3.367907000	0.139508000	0.662390000	6	2.571747000	0.029934000	0.975092000
6	4.510751000	-0.674251000	0.665749000	6	3.807300000	-0.696164000	1.183734000
6	4.727299000	-1.596768000	-0.349812000	6	4.662624000	-0.957187000	0.131364000
6	3.814116000	-1.716087000	-1.403407000	6	4.339636000	-0.510055000	-1.159427000
6	2.663722000	-0.936225000	-1.400480000	6	3.140898000	0.205941000	-1.393445000
8	3.250873000	1.059935000	1.637100000	8	1.791602000	0.277869000	1.938119000
1	2.502116000	1.663562000	1.381798000	1	1.588375000	3.157094000	0.086550000

1	4.003363000	-2.400536000	-2.223620000	1	5.011396000	-0.712338000	-1.986848000
6	-0.602959000	-0.842478000	-1.226926000	6	-0.622283000	-0.592274000	-1.254386000
6	-1.481596000	-1.522928000	-0.191715000	6	-1.135917000	-1.635694000	-0.276864000
6	-2.838598000	-1.779314000	-0.446607000	6	-1.805374000	-2.762132000	-0.786407000
6	-3.631843000	-2.428026000	0.505752000	6	-2.295714000	-3.751280000	0.068383000
6	-3.077707000	-2.825767000	1.720299000	6	-2.116650000	-3.627550000	1.447011000
6	-1.730172000	-2.581038000	1.990225000	6	-1.448015000	-2.520762000	1.967170000
6	-0.947588000	-1.937450000	1.033318000	6	-0.962839000	-1.533735000	1.104891000
1	-1.153354000	-0.695959000	-2.157695000	1	-1.373815000	-0.426219000	-2.036219000
1	0.250878000	-1.479870000	-1.443154000	1	0.270807000	-0.960329000	-1.769442000
1	-1.292891000	-2.890914000	2.933562000	1	-1.302570000	-2.420256000	3.037941000
8	-3.349392000	-1.371298000	-1.653217000	8	-1.943027000	-2.836678000	-2.149991000
1	-4.290608000	-1.588014000	-1.691177000	1	-2.446036000	-3.630812000	-2.374591000
1	-2.881759000	1.062027000	-1.278603000	1	-3.153778000	0.701396000	-0.495949000
1	-0.024490000	4.402181000	1.174079000	1	-0.631337000	4.905178000	0.628878000
1	-2.434436000	4.865779000	0.712405000	1	-3.128310000	4.843939000	0.721045000
1	-3.828377000	3.224876000	-0.493889000	1	-4.364880000	2.787828000	0.152291000
1	5.226683000	-0.537929000	1.469446000	1	4.023297000	-1.027573000	2.194401000
1	5.625618000	-2.207128000	-0.335729000	1	5.585111000	-1.506143000	0.294386000
1	1.987323000	-0.997208000	-2.245863000	1	2.909568000	0.550483000	-2.397307000
1	-4.681496000	-2.616907000	0.292056000	1	-2.814255000	-4.613106000	-0.346673000
1	-3.701922000	-3.327790000	2.453292000	1	-2.500779000	-4.399459000	2.107402000
1	0.102898000	-1.746860000	1.236544000	1	-0.437466000	-0.673026000	1.505132000
V							
6	2.022751000	1.937969000	0.343406000				
6	0.681924000	1.636161000	0.102080000				
6	-0.191803000	2.607757000	-0.410514000				
6	0.235735000	3.889434000	-0.746753000				
6	1.583004000	4.179396000	-0.533017000				
6	2.454224000	3.223623000	0.013171000				
7	-0.071011000	0.473208000	0.322862000				
7	-1.436554000	2.004374000	-0.454546000				
6	-1.376825000	0.712937000	-0.003844000				
6	-2.564959000	-0.084792000	0.107862000				
6	-2.585289000	-1.524551000	-0.149824000				
6	-3.898108000	-2.136231000	-0.098718000				
6	-5.041894000	-1.426558000	0.163088000				
6	-4.998282000	-0.029257000	0.406237000				
6	-3.782639000	0.612364000	0.365278000				
8	-1.558829000	-2.203546000	-0.427195000				
1	-5.903589000	0.519057000	0.644437000				
6	0.475529000	-0.723936000	1.000938000				
6	1.640307000	-1.350577000	0.255809000				
6	2.920415000	-1.412795000	0.830835000				
6	3.977048000	-2.037037000	0.160518000				
6	3.764749000	-2.606291000	-1.093476000				
6	2.498815000	-2.555539000	-1.678680000				
6	1.448857000	-1.936991000	-1.001237000				
1	0.777965000	-0.422499000	2.005475000				
1	-0.337825000	-1.446063000	1.043730000				
1	2.326112000	-3.006553000	-2.650669000				
8	3.101587000	-0.819800000	2.058506000				
1	4.007939000	-0.977849000	2.354958000				
1	2.694979000	1.220127000	0.794048000				
1	-0.449609000	4.627860000	-1.148964000				
1	1.960258000	5.165760000	-0.782678000				
1	3.491546000	3.490372000	0.188051000				
1	-3.927215000	-3.205481000	-0.286704000				
1	-5.999479000	-1.942123000	0.192355000				
1	-3.742138000	1.674032000	0.600370000				
1	4.961394000	-2.071131000	0.622818000				
1	4.590243000	-3.090348000	-1.607180000				
1	0.445839000	-1.924756000	-1.414434000				
1	-2.266898000	2.382775000	-0.883275000				
F							
I				II			

6	-2.236981000	2.071826000	-0.871402000	6	-2.193064000	1.909457000	-1.059995000
6	-0.927456000	1.817744000	-0.452541000	6	-0.873395000	1.673563000	-0.664605000
6	-0.171312000	2.770029000	0.270962000	6	-0.095027000	2.672282000	-0.039195000
6	-0.728887000	4.015715000	0.587163000	6	-0.632061000	3.940688000	0.211893000
6	-2.030902000	4.274545000	0.172611000	6	-1.945952000	4.177478000	-0.176559000
6	-2.772726000	3.315725000	-0.546162000	6	-2.712773000	3.176396000	-0.804616000
7	-0.085059000	0.725240000	-0.607227000	7	-0.045771000	0.556941000	-0.767964000
7	1.089869000	2.271721000	0.548993000	7	1.168767000	2.178005000	0.215452000
6	1.108320000	1.067561000	0.022331000	6	1.179247000	0.929961000	-0.235200000
6	2.304503000	0.196786000	0.009024000	6	2.392687000	0.111372000	-0.150441000
6	2.920612000	-0.223362000	1.205508000	6	3.330954000	0.403927000	0.879395000
6	4.093827000	-0.984250000	1.156505000	6	4.473834000	-0.397334000	1.020509000
6	4.666282000	-1.343583000	-0.062673000	6	4.737250000	-1.435731000	0.135253000
6	4.045305000	-0.932888000	-1.233248000	6	3.849218000	-1.663098000	-0.911288000
6	2.884853000	-0.171916000	-1.212644000	6	2.699096000	-0.910854000	-1.069054000
8	2.331113000	0.109307000	2.390073000	8	3.174741000	1.429619000	1.739555000
1	2.922596000	-0.136848000	3.113873000	1	2.444667000	2.001784000	1.382143000
9	4.588084000	-1.269663000	-2.426629000	9	4.121903000	-2.639344000	-1.811286000
6	-0.459819000	-0.568401000	-1.183359000	6	-0.518311000	-0.800125000	-1.081591000
6	-1.569643000	-1.280066000	-0.433973000	6	-1.686984000	-1.263320000	-0.235135000
6	-1.372780000	-1.701465000	0.894122000	6	-1.543976000	-1.411407000	1.157155000
6	-2.396655000	-2.345288000	1.593333000	6	-2.611133000	-1.872287000	1.932745000
6	-3.623982000	-2.594312000	0.977316000	6	-3.826772000	-2.207048000	1.335582000
6	-3.800082000	-2.196560000	-0.339707000	6	-3.951894000	-2.073954000	-0.039542000
6	-2.795355000	-1.546572000	-1.047224000	6	-2.904315000	-1.609228000	-0.825707000
1	-0.765598000	-0.415343000	-2.223725000	1	-0.790476000	-0.849415000	-2.140998000
1	0.441675000	-1.183582000	-1.191776000	1	0.321601000	-1.473878000	-0.920386000
9	-4.981177000	-2.441078000	-0.954523000	9	-5.122054000	-2.401796000	-0.634573000
8	-0.140201000	-1.467809000	1.431208000	8	-0.325708000	-1.097972000	1.688451000
1	-0.149099000	-1.613378000	2.385760000	1	-0.338954000	-1.228839000	2.645881000
1	-2.819728000	1.339533000	-1.418347000	1	-2.795388000	1.148268000	-1.540663000
1	-0.149268000	4.748763000	1.139094000	1	-0.030378000	4.706995000	0.689561000
1	-2.488031000	5.231856000	0.404817000	1	-2.390846000	5.151498000	0.002598000
1	-3.787223000	3.550676000	-0.854290000	1	-3.734278000	3.396284000	-1.099445000
1	4.560807000	-1.306316000	2.084109000	1	5.159721000	-0.166220000	1.828160000
1	5.575699000	-1.932241000	-0.107563000	1	5.627274000	-2.048041000	0.230717000
1	2.450808000	0.158610000	-2.150003000	1	2.072944000	-1.101339000	-1.931783000
1	-2.231644000	-2.662172000	2.619815000	1	-2.490690000	-1.977598000	3.007862000
1	-4.428825000	-3.093483000	1.505135000	1	-4.662752000	-2.568362000	1.923775000
1	-2.978031000	-1.247718000	-2.074773000	1	-3.044104000	-1.523098000	-1.898786000
		III				IV	
6	-2.421002000	1.891080000	-0.936464000	6	-2.499364000	2.142308000	-0.303341000
6	-1.093478000	1.726039000	-0.533310000	6	-1.115790000	1.977005000	-0.318506000
6	-0.431616000	2.668331000	0.282224000	6	-0.247387000	3.035116000	0.043185000
6	-1.098231000	3.820630000	0.717194000	6	-0.743642000	4.276219000	0.437470000
6	-2.419323000	3.992302000	0.318913000	6	-2.141416000	4.435808000	0.460523000
6	-3.068901000	3.042614000	-0.495441000	6	-2.997143000	3.397666000	0.095720000
7	-0.164119000	0.720209000	-0.785628000	7	-0.327966000	0.894847000	-0.679423000
7	0.863804000	2.247581000	0.509918000	7	1.036157000	2.570480000	-0.140181000
6	1.004503000	1.097168000	-0.139316000	6	1.030899000	1.267960000	-0.651354000
6	2.281495000	0.374496000	-0.119907000	6	2.147343000	0.310240000	-0.371680000
6	3.147378000	0.571060000	0.993855000	6	2.359570000	-0.217105000	0.968957000
6	4.347013000	-0.153222000	1.072546000	6	3.474323000	-1.122838000	1.176150000
6	4.735975000	-1.012421000	0.052804000	6	4.312142000	-1.477791000	0.141951000
6	3.917393000	-1.135824000	-1.066011000	6	4.065879000	-0.936634000	-1.125666000
6	2.715508000	-0.459689000	-1.169213000	6	3.002332000	-0.050430000	-1.387812000
8	2.870364000	1.431372000	1.992079000	8	1.599484000	0.109428000	1.920698000
1	2.107242000	1.993707000	1.691969000	1	1.872508000	3.052395000	0.143257000
9	4.312466000	-1.929234000	-2.091019000	9	4.875252000	-1.270575000	-2.139875000
6	-0.537943000	-0.586757000	-1.345785000	6	-0.838671000	-0.300557000	-1.323792000
6	-1.491443000	-1.367744000	-0.457906000	6	-1.517197000	-1.282829000	-0.384502000
6	-2.779412000	-1.709696000	-0.902137000	6	-2.332281000	-2.287632000	-0.936975000
6	-3.638097000	-2.452028000	-0.084303000	6	-2.973817000	-3.216278000	-0.115928000
6	-3.228550000	-2.861918000	1.182377000	6	-2.811280000	-3.159551000	1.269272000
6	-1.952861000	-2.518747000	1.612226000	6	-1.999698000	-2.168086000	1.800650000
6	-1.086374000	-1.786804000	0.813655000	6	-1.352255000	-1.233060000	1.000493000
1	-0.982465000	-0.427795000	-2.329441000	1	-1.544993000	-0.008923000	-2.110842000

1	0.372341000	-1.165643000	-1.481835000	1	-0.001888000	-0.786016000	-1.835917000
9	-1.542321000	-2.913982000	2.838144000	9	-1.832889000	-2.107684000	3.142825000
8	-3.155295000	-1.286262000	-2.152830000	8	-2.451117000	-2.304843000	-2.304982000
1	-4.060419000	-1.574094000	-2.331180000	1	-3.065322000	-3.004695000	-2.563343000
1	-2.924498000	1.161906000	-1.561767000	1	-3.167163000	1.331480000	-0.569953000
1	-0.590228000	4.548572000	1.341112000	1	-0.077671000	5.087146000	0.712185000
1	-2.963869000	4.875569000	0.638450000	1	-2.557207000	5.390530000	0.767178000
1	-4.100570000	3.213467000	-0.787781000	1	-4.070990000	3.553681000	0.122283000
1	4.974005000	-0.002544000	1.944422000	1	3.617371000	-1.512624000	2.178331000
1	5.669229000	-1.562615000	0.102019000	1	5.148226000	-2.155596000	0.275265000
1	2.150694000	-0.552801000	-2.088204000	1	2.873188000	0.336474000	-2.393190000
1	-4.633452000	-2.707875000	-0.438841000	1	-3.602333000	-3.985987000	-0.557384000
1	-3.882480000	-3.436390000	1.828639000	1	-3.302345000	-3.869606000	1.925170000
1	-0.097392000	-1.543141000	1.188281000	1	-0.717779000	-0.480573000	1.455591000
V							
6	1.898312000	2.348762000	0.326897000				
6	0.620315000	1.859072000	0.055810000				
6	-0.332867000	2.658843000	-0.593468000				
6	-0.044484000	3.945945000	-1.038930000				
6	1.243067000	4.422295000	-0.794126000				
6	2.189948000	3.640906000	-0.113085000				
7	0.002023000	0.636217000	0.358187000				
7	-1.491484000	1.902298000	-0.633496000				
6	-1.301123000	0.676992000	-0.054505000				
6	-2.383705000	-0.254962000	0.077232000				
6	-2.205686000	-1.700555000	-0.031268000				
6	-3.432083000	-2.476416000	0.002491000				
6	-4.675609000	-1.911493000	0.119838000				
6	-4.793341000	-0.508926000	0.213707000				
6	-3.695002000	0.303792000	0.185033000				
8	-1.086597000	-2.265989000	-0.174394000				
9	-6.032715000	0.031853000	0.366010000				
6	0.641860000	-0.404882000	1.191760000				
6	1.915835000	-0.960593000	0.580547000				
6	3.156682000	-0.799502000	1.220895000				
6	4.317030000	-1.360050000	0.678646000				
6	4.258760000	-2.086925000	-0.508746000				
6	3.027028000	-2.240562000	-1.132165000				
6	1.863157000	-1.699845000	-0.606529000				
1	0.847479000	0.036086000	2.168648000				
1	-0.080392000	-1.214440000	1.278389000				
9	2.961733000	-2.950507000	-2.283269000				
8	3.189035000	-0.054239000	2.376948000				
1	4.085871000	-0.064689000	2.736727000				
1	2.624824000	1.768447000	0.879810000				
1	-0.789278000	4.549699000	-1.546162000				
1	1.512184000	5.419693000	-1.126100000				
1	3.175299000	4.051843000	0.081536000				
1	-3.312332000	-3.552172000	-0.076171000				
1	-5.575905000	-2.518455000	0.149099000				
1	-3.839433000	1.371991000	0.316495000				
1	5.269823000	-1.223953000	1.184771000				
1	5.148006000	-2.528445000	-0.944678000				
1	0.903070000	-1.876539000	-1.078804000				
1	-2.332227000	2.129668000	-1.141683000				
Cl							
I				II			
6	-2.044806000	2.227193000	-1.086109000	6	-2.112406000	2.033819000	-1.212250000
6	-0.767452000	1.971175000	-0.578358000	6	-0.802731000	1.844822000	-0.762360000
6	0.014062000	2.978933000	0.034468000	6	-0.040412000	2.905447000	-0.225957000
6	-0.484294000	4.283512000	0.146543000	6	-0.583452000	4.191730000	-0.121453000
6	-1.754127000	4.544251000	-0.356630000	6	-1.887374000	4.382402000	-0.564446000
6	-2.521575000	3.530143000	-0.964220000	6	-2.638281000	3.319006000	-1.103202000
7	0.017870000	0.826913000	-0.539178000	7	0.027325000	0.725259000	-0.728109000
7	1.235263000	2.463944000	0.434124000	7	1.216777000	2.443216000	0.109684000
6	1.205498000	1.197171000	0.085631000	6	1.238510000	1.155029000	-0.207458000

6	2.350004000	0.273776000	0.249303000	6	2.449674000	0.354052000	-0.000633000
6	2.903311000	0.005826000	1.518098000	6	3.354012000	0.757919000	1.022498000
6	4.030039000	-0.816568000	1.626160000	6	4.487075000	-0.024963000	1.287950000
6	4.614619000	-1.385380000	0.497177000	6	4.776275000	-1.150543000	0.527917000
6	4.061462000	-1.126572000	-0.753449000	6	3.929450000	-1.496104000	-0.526739000
6	2.946154000	-0.303058000	-0.880112000	6	2.786362000	-0.756088000	-0.795837000
8	2.302675000	0.544691000	2.613577000	8	3.173174000	1.869501000	1.756177000
1	2.846885000	0.365099000	3.392388000	1	2.450581000	2.395859000	1.317379000
17	4.787312000	-1.833344000	-2.194998000	17	4.322962000	-2.882218000	-1.544165000
6	-0.406955000	-0.516598000	-0.937671000	6	-0.439828000	-0.657260000	-0.911393000
6	-1.588378000	-1.051334000	-0.151143000	6	-1.612842000	-1.037724000	-0.030062000
6	-1.483746000	-1.264720000	1.235928000	6	-1.472403000	-1.066877000	1.369934000
6	-2.573840000	-1.752149000	1.960792000	6	-2.544746000	-1.450788000	2.179085000
6	-3.773521000	-2.051199000	1.315756000	6	-3.761141000	-1.824457000	1.609544000
6	-3.869484000	-1.861684000	-0.059292000	6	-3.894094000	-1.812631000	0.224328000
6	-2.789655000	-1.364463000	-0.788260000	6	-2.831654000	-1.423379000	-0.590064000
1	-0.656804000	-0.507052000	-2.003929000	1	-0.706059000	-0.808668000	-1.962457000
1	0.455734000	-1.172684000	-0.808249000	1	0.399356000	-1.311401000	-0.681438000
17	-5.374024000	-2.246805000	-0.892048000	17	-5.420460000	-2.291815000	-0.512422000
8	-0.278248000	-0.990933000	1.806020000	8	-0.255406000	-0.719293000	1.874054000
1	-0.334806000	-1.042710000	2.768919000	1	-0.267161000	-0.773483000	2.839136000
1	-2.647775000	1.454801000	-1.549595000	1	-2.702772000	1.225257000	-1.625610000
1	0.115243000	5.058225000	0.613707000	1	0.006056000	5.005405000	0.288081000
1	-2.165826000	5.546387000	-0.282474000	1	-2.337199000	5.368309000	-0.498845000
1	-3.509698000	3.768585000	-1.346302000	1	-3.652398000	3.504119000	-1.443634000
1	4.451278000	-1.020767000	2.607454000	1	5.146980000	0.288592000	2.089550000
1	5.487184000	-2.021557000	0.592148000	1	5.662450000	-1.740669000	0.732830000
1	2.548581000	-0.082891000	-1.864618000	1	2.178912000	-1.021841000	-1.651768000
1	-2.484202000	-1.907689000	3.032779000	1	-2.428547000	-1.465654000	3.259744000
1	-4.618519000	-2.431566000	1.878098000	1	-4.591536000	-2.124214000	2.238434000
1	-2.884411000	-1.219227000	-1.859621000	1	-2.952006000	-1.424506000	-1.668706000
		III				IV	
6	-2.564057000	2.065006000	-1.081609000	6	-2.322140000	2.039962000	-1.383986000
6	-1.288657000	1.933174000	-0.526375000	6	-1.163068000	1.853204000	-0.633450000
6	-0.801746000	2.817996000	0.458970000	6	-0.748206000	2.813080000	0.317756000
6	-1.596791000	3.876294000	0.916200000	6	-1.476449000	3.978223000	0.534504000
6	-2.866595000	4.014511000	0.367084000	6	-2.636857000	4.171844000	-0.233860000
6	-3.341244000	3.123195000	-0.616418000	6	-3.048531000	3.223851000	-1.171092000
7	-0.265809000	1.015726000	-0.754857000	7	-0.243056000	0.811587000	-0.607382000
7	0.482478000	2.450600000	0.809923000	7	0.420926000	2.340188000	0.892216000
6	0.784225000	1.388579000	0.071771000	6	0.661174000	1.014476000	0.462910000
6	2.098003000	0.744430000	0.187168000	6	2.073589000	0.541214000	0.340961000
6	2.799373000	0.867546000	1.421119000	6	2.586652000	-0.528065000	1.212315000
6	4.024989000	0.204179000	1.584970000	6	3.988704000	-0.881431000	1.069988000
6	4.597850000	-0.513697000	0.544254000	6	4.809184000	-0.281596000	0.142412000
6	3.947012000	-0.560886000	-0.690485000	6	4.270936000	0.713839000	-0.687584000
6	2.720481000	0.061233000	-0.874219000	6	2.919853000	1.112883000	-0.582345000
8	2.340866000	1.599118000	2.449810000	8	1.863917000	-1.136047000	2.036197000
1	1.579490000	2.143174000	2.109595000	1	0.754728000	2.665371000	1.787276000
17	4.699457000	-1.407186000	-2.042191000	17	5.293900000	1.491708000	-1.864904000
6	-0.472825000	-0.242484000	-1.486514000	6	-0.487496000	-0.505214000	-1.192364000
6	-1.466485000	-1.173395000	-0.811533000	6	-1.548098000	-1.320362000	-0.467978000
6	-2.665875000	-1.535797000	-1.446134000	6	-2.745138000	-1.672724000	-1.113546000
6	-3.556399000	-2.417958000	-0.825638000	6	-3.723810000	-2.411810000	-0.442166000
6	-3.266846000	-2.946848000	0.428957000	6	-3.525485000	-2.805945000	0.878382000
6	-2.076797000	-2.586887000	1.058654000	6	-2.338052000	-2.455606000	1.517030000
6	-1.183069000	-1.713232000	0.446634000	6	-1.353371000	-1.723686000	0.858352000
1	-0.806635000	-0.004332000	-2.497606000	1	-0.773848000	-0.367007000	-2.235695000
1	0.486989000	-0.747328000	-1.565267000	1	0.465341000	-1.039990000	-1.187767000
17	-1.695713000	-3.252020000	2.643281000	17	-2.079773000	-2.955599000	3.188621000
8	-2.922965000	-0.994178000	-2.677264000	8	-2.912839000	-1.272310000	-2.414920000
1	-3.776975000	-1.312431000	-2.999536000	1	-3.786685000	-1.546921000	-2.723104000
1	-2.933423000	1.380718000	-1.837585000	1	-2.661552000	1.290211000	-2.089474000
1	-1.223051000	4.560190000	1.671041000	1	-1.162608000	4.707208000	1.274397000
1	-3.507473000	4.825541000	0.699055000	1	-3.224540000	5.072309000	-0.085991000
1	-4.337874000	3.265709000	-1.023035000	1	-3.955084000	3.395350000	-1.743149000
1	4.525501000	0.289095000	2.543330000	1	4.359057000	-1.660190000	1.727587000

1	5.551653000	-1.011466000	0.678243000	1	5.851521000	-0.563418000	0.043371000
1	2.270861000	0.052474000	-1.858877000	1	2.553125000	1.897852000	-1.235377000
1	-4.482598000	-2.691521000	-1.324779000	1	-4.645694000	-2.679603000	-0.952825000
1	-3.957727000	-3.629770000	0.909772000	1	-4.283501000	-3.378183000	1.400910000
1	-0.259560000	-1.447127000	0.950145000	1	-0.434685000	-1.458709000	1.373827000
V							
6	1.786963000	2.689788000	0.275541000				
6	0.581691000	2.049503000	-0.015571000				
6	-0.391572000	2.675201000	-0.809548000				
6	-0.192466000	3.929752000	-1.379787000				
6	1.024266000	4.555070000	-1.111634000				
6	1.988551000	3.949284000	-0.289855000				
7	0.057190000	0.811465000	0.386725000				
7	-1.469130000	1.805814000	-0.835135000				
6	-1.209394000	0.678619000	-0.107081000				
6	-2.209956000	-0.337740000	0.071483000				
6	-1.892356000	-1.762908000	0.124468000				
6	-3.042861000	-2.642840000	0.190217000				
6	-4.336683000	-2.191321000	0.196072000				
6	-4.597888000	-0.801233000	0.137507000				
6	-3.565523000	0.100481000	0.068689000				
8	-0.720095000	-2.224033000	0.090640000				
17	-6.270743000	-0.231213000	0.193460000				
6	0.732656000	-0.072614000	1.361106000				
6	2.089229000	-0.559030000	0.881470000				
6	3.265114000	-0.219304000	1.571199000				
6	4.504261000	-0.710466000	1.149410000				
6	4.589375000	-1.542358000	0.036435000				
6	3.421822000	-1.879553000	-0.645137000				
6	2.181834000	-1.405633000	-0.229176000				
1	0.834795000	0.483364000	2.294523000				
1	0.082460000	-0.934492000	1.497241000				
17	3.520433000	-2.937937000	-2.052100000				
8	3.154554000	0.624178000	2.648056000				
1	4.023569000	0.740321000	3.055298000				
1	2.522803000	2.246503000	0.933050000				
1	-0.951383000	4.399246000	-1.996258000				
1	1.223487000	5.533324000	-1.536666000				
1	2.915141000	4.475222000	-0.083987000				
1	-2.825528000	-3.705426000	0.230742000				
1	-5.167299000	-2.888405000	0.250525000				
1	-3.792458000	1.162226000	0.074171000				
1	5.406765000	-0.437092000	1.690630000				
1	5.549598000	-1.922187000	-0.293562000				
1	1.266652000	-1.707059000	-0.726231000				
1	-2.287792000	1.891680000	-1.418140000				
Br							
I				II			
6	-1.876252000	2.388807000	-1.395413000	6	-1.996762000	2.291020000	-1.367619000
6	-0.633056000	2.210802000	-0.780894000	6	-0.692720000	2.172341000	-0.878379000
6	0.147379000	3.301964000	-0.331415000	6	0.057568000	3.300770000	-0.481194000
6	-0.316520000	4.613170000	-0.499192000	6	-0.491347000	4.586183000	-0.561674000
6	-1.551833000	4.796854000	-1.110856000	6	-1.789479000	4.707324000	-1.044347000
6	-2.319089000	3.699941000	-1.552253000	6	-2.528903000	3.576003000	-1.442097000
7	0.115143000	1.080180000	-0.482121000	7	0.140483000	1.072751000	-0.677226000
7	1.331957000	2.849391000	0.224191000	7	1.310128000	2.896060000	-0.064543000
6	1.281613000	1.540027000	0.122691000	6	1.340833000	1.576508000	-0.200099000
6	2.390053000	0.642187000	0.516513000	6	2.548873000	0.817983000	0.140489000
6	2.870884000	0.600853000	1.841284000	6	3.433209000	1.365319000	1.113706000
6	3.969074000	-0.206142000	2.158641000	6	4.561592000	0.631663000	1.508897000
6	4.594019000	-0.982171000	1.185349000	6	4.865844000	-0.589233000	0.921370000
6	4.111526000	-0.947580000	-0.119062000	6	4.040142000	-1.082841000	-0.089344000
6	3.027104000	-0.142539000	-0.454281000	6	2.902171000	-0.392831000	-0.481939000
8	2.228809000	1.339166000	2.786649000	8	3.237933000	2.568722000	1.678690000
1	2.724755000	1.291080000	3.615318000	1	2.521839000	3.024674000	1.157592000
35	4.956067000	-2.003866000	-1.473270000	35	4.488976000	-2.728817000	-0.960726000

6	-0.328604000	-0.305940000	-0.642722000	6	-0.314831000	-0.325492000	-0.680586000
6	-1.555704000	-0.663250000	0.174097000	6	-1.522312000	-0.587277000	0.197114000
6	-1.516346000	-0.618504000	1.580186000	6	-1.450881000	-0.388193000	1.588507000
6	-2.649484000	-0.941654000	2.330794000	6	-2.555608000	-0.663409000	2.398634000
6	-3.828660000	-1.330597000	1.695717000	6	-3.735762000	-1.152888000	1.840697000
6	-3.860723000	-1.396560000	0.306913000	6	-3.800378000	-1.365924000	0.467539000
6	-2.737755000	-1.064962000	-0.449329000	6	-2.706129000	-1.086517000	-0.348709000
1	-0.535281000	-0.489857000	-1.702358000	1	-0.536306000	-0.629131000	-1.708980000
1	0.510422000	-0.942429000	-0.355869000	1	0.516173000	-0.932589000	-0.325157000
35	-5.466313000	-1.942386000	-0.581158000	35	-5.409630000	-2.046065000	-0.312234000
8	-0.328885000	-0.268603000	2.145851000	8	-0.267196000	0.066310000	2.086264000
1	-0.429641000	-0.126959000	3.096059000	1	-0.326842000	0.166229000	3.045970000
1	-2.477953000	1.552664000	-1.732617000	1	-2.578730000	1.429980000	-1.672615000
1	0.282527000	5.451692000	-0.158737000	1	0.089252000	5.451442000	-0.259083000
1	-1.936059000	5.802470000	-1.253585000	1	-2.243678000	5.690466000	-1.120518000
1	-3.279697000	3.879759000	-2.025690000	1	-3.538943000	3.707016000	-1.817922000
1	4.335549000	-0.234271000	3.181984000	1	5.205436000	1.057653000	2.270777000
1	5.443014000	-1.604502000	1.443570000	1	5.748080000	-1.140190000	1.226474000
1	2.683698000	-0.100525000	-1.481798000	1	2.310630000	-0.779595000	-1.301850000
1	-2.609790000	-0.898486000	3.416191000	1	-2.492981000	-0.501710000	3.471770000
1	-4.706023000	-1.583008000	2.279661000	1	-4.590160000	-1.367216000	2.471890000
1	-2.781728000	-1.118517000	-1.532251000	1	-2.771787000	-1.261126000	-1.417775000
	III				IV		
6	2.502134000	2.763224000	1.132352000	6	2.809889000	2.785544000	0.488639000
6	1.368590000	2.455706000	0.375583000	6	1.482185000	2.611974000	0.103857000
6	1.086337000	3.095351000	-0.849997000	6	0.863703000	3.495572000	-0.813165000
6	1.948929000	4.077540000	-1.352317000	6	1.562133000	4.564649000	-1.370834000
6	3.078307000	4.389558000	-0.603869000	6	2.904991000	4.730789000	-0.986713000
6	3.348979000	3.742704000	0.618919000	6	3.512645000	3.866044000	-0.077477000
7	0.328289000	1.553007000	0.583820000	7	0.531835000	1.679682000	0.491766000
7	-0.095594000	2.599134000	-1.363822000	7	-0.446491000	3.088219000	-0.929299000
6	-0.532931000	1.695322000	-0.494486000	6	-0.715805000	2.007244000	-0.080609000
6	-1.789982000	0.975776000	-0.731109000	6	-1.754879000	0.987017000	-0.425908000
6	-2.225566000	0.810656000	-2.077773000	6	-1.566903000	0.076218000	-1.547474000
6	-3.386801000	0.067568000	-2.339595000	6	-2.630564000	-0.866222000	-1.840069000
6	-4.155167000	-0.450862000	-1.306336000	6	-3.782761000	-0.905357000	-1.086922000
6	-3.770405000	-0.213340000	0.014314000	6	-3.925548000	-0.007056000	-0.015897000
6	-2.611450000	0.491405000	0.303896000	6	-2.917924000	0.932523000	0.307004000
8	-1.572390000	1.340913000	-3.124247000	8	-0.517180000	0.109777000	-2.241722000
1	-0.900330000	1.979539000	-2.760482000	1	-1.115782000	3.465133000	-1.578857000
35	-4.864602000	-0.853388000	1.450004000	35	-5.506124000	-0.046810000	1.023074000
6	0.392449000	0.481196000	1.588490000	6	0.710438000	0.747753000	1.588370000
6	1.508150000	-0.517516000	1.330078000	6	1.567993000	-0.462116000	1.256647000
6	2.560727000	-0.682673000	2.245266000	6	2.068549000	-1.240981000	2.315430000
6	3.564066000	-1.627885000	2.007422000	6	2.860789000	-2.361983000	2.062215000
6	3.534088000	-2.415164000	0.859938000	6	3.162455000	-2.725368000	0.749882000
6	2.490158000	-2.251475000	-0.047753000	6	2.659694000	-1.957422000	-0.294786000
6	1.485653000	-1.317537000	0.183441000	6	1.868136000	-0.834948000	-0.054299000
1	0.513792000	0.936700000	2.572520000	1	1.153056000	1.277805000	2.440944000
1	-0.559626000	-0.043881000	1.582035000	1	-0.282760000	0.425952000	1.916975000
35	2.434706000	-3.329315000	-1.626375000	35	3.066546000	-2.451729000	-2.100220000
8	2.563920000	0.109439000	3.362045000	8	1.735670000	-0.845447000	3.583732000
1	3.344621000	-0.088515000	3.896720000	1	2.161168000	-1.429911000	4.225339000
1	2.715228000	2.267398000	2.073059000	1	3.289540000	2.105129000	1.182485000
1	1.731451000	4.573124000	-2.292669000	1	1.086472000	5.242782000	-2.071174000
1	3.766714000	5.147580000	-0.964698000	1	3.475171000	5.552810000	-1.408032000
1	4.239947000	4.015951000	1.176118000	1	4.550308000	4.022310000	0.199815000
1	-3.681393000	-0.068649000	-3.374470000	1	-2.480017000	-1.543508000	-2.674379000
1	-5.057210000	-1.012036000	-1.521582000	1	-4.575405000	-1.612221000	-1.304868000
1	-2.370498000	0.702391000	1.337716000	1	-3.067246000	1.614840000	1.136740000
1	4.374156000	-1.747823000	2.722490000	1	3.244374000	-2.953893000	2.889845000
1	4.313149000	-3.145865000	0.676962000	1	3.779255000	-3.593736000	0.549863000
1	0.677655000	-1.206424000	-0.531900000	1	1.476643000	-0.252227000	-0.880211000
				V			
6	1.680561000	3.168853000	0.127760000				
6	0.565003000	2.372865000	-0.134943000				

6	-0.412195000	2.784916000	-1.053822000
6	-0.300595000	3.969500000	-1.777113000
6	0.829074000	4.749307000	-1.534159000
6	1.793512000	4.359601000	-0.590585000
7	0.140844000	1.148026000	0.402413000
7	-1.394880000	1.809778000	-1.017224000
6	-1.071859000	0.819164000	-0.133068000
6	-1.973742000	-0.269660000	0.126522000
6	-1.515055000	-1.633744000	0.380475000
6	-2.570381000	-2.621064000	0.499084000
6	-3.901375000	-2.317214000	0.380679000
6	-4.299753000	-0.982559000	0.130913000
6	-3.363963000	0.012265000	-0.001651000
8	-0.301786000	-1.961629000	0.468135000
35	-6.169564000	-0.565167000	0.019465000
6	0.830133000	0.478333000	1.526594000
6	2.259935000	0.084415000	1.199502000
6	3.341917000	0.635327000	1.906536000
6	4.651720000	0.229001000	1.633586000
6	4.901087000	-0.725140000	0.650787000
6	3.827247000	-1.269456000	-0.049329000
6	2.519408000	-0.882694000	0.221470000
1	0.808268000	1.159968000	2.378634000
1	0.261199000	-0.423519000	1.743391000
35	4.160815000	-2.586522000	-1.399566000
8	3.068201000	1.595013000	2.848208000
1	3.889130000	1.859585000	3.284717000
1	2.413223000	2.893918000	0.874572000
1	-1.060015000	4.271700000	-2.490082000
1	0.958375000	5.679886000	-2.076918000
1	2.649185000	5.002273000	-0.410857000
1	-2.248121000	-3.640621000	0.686183000
1	-4.656223000	-3.090590000	0.481803000
1	-3.697976000	1.034895000	-0.146999000
1	5.479996000	0.663687000	2.187952000
1	5.916279000	-1.038915000	0.436898000
1	1.677710000	-1.341354000	-0.284836000
1	-2.181963000	1.734402000	-1.643430000

Table S2. Mulliken Population Analysis of Atomic Charges in the O_1–H_1···N_1 Bond of Conformers III in S_0 and S_1 States for Different Substituents and Solvents.

III (S_0)	H		F		Cl		Br	
Solvent	O_1	N_1	O_1	N_1	O_1	N_1	O_1	N_1
n-Hexane	-0.584	-0.661	-0.585	-0.660	-0.579	-0.661	-0.579	-0.661
1,4-Dioxane	-0.586	-0.662	-0.587	-0.661	-0.581	-0.662	-0.581	-0.662
Ethanol	-0.598	-0.671	-0.599	-0.670	-0.593	-0.670	-0.593	-0.670
Acetonitrile	-0.599	-0.672	-0.600	-0.670	-0.593	-0.670	-0.593	-0.671
Water	-0.599	-0.672	-0.600	-0.671	-0.594	-0.671	-0.594	-0.671

III (S_1)	H		F		Cl		Br	
Solvent	O_1	N_1	O_1	N_1	O_1	N_1	O_1	N_1
n-Hexane	-0.516	-0.684	-0.511	-0.685	-0.513	-0.685	-0.514	-0.686
1,4-Dioxane	-0.518	-0.685	-0.514	-0.686	-0.515	-0.686	-0.516	-0.687
Ethanol	-0.535	-0.691	-0.529	-0.692	-0.531	-0.692	-0.531	-0.693
Acetonitrile	-0.536	-0.691	-0.530	-0.693	-0.532	-0.692	-0.532	-0.693
Water	-0.537	-0.691	-0.530	-0.693	-0.532	-0.692	-0.533	-0.693
